# Heart rate and gas exchange dynamic responses to multiple brief exercise bouts (MBEB) in early‐ and late‐pubertal boys and girls

**DOI:** 10.14814/phy2.15397

**Published:** 2022-08-03

**Authors:** Ronen Bar‐Yoseph, Shlomit Radom‐Aizik, Nicholas Coronato, Nazanin Moradinasab, Thomas J. Barstow, Annamarie Stehli, Don Brown, Dan M. Cooper

**Affiliations:** ^1^ Pediatric Exercise and Genomics Research Center University of California at Irvine Irvine California USA; ^2^ Pediatric Pulmonary Division Ruth Children's Hospital, Rambam Health Care Center Haifa Israel; ^3^ University of Virginia Charlottesville Virginia USA; ^4^ United States Military Academy West Point New York USA; ^5^ Kansas State University Manhattan Kansas USA; ^6^ Department of Pediatrics, Institute for Clinical and Translational Science, and Pediatric Exercise and Genomics Research Center University of California Irvine California USA

**Keywords:** adolescents, cardiopulmonary exercise testing, children, puberty

## Abstract

Natural patterns of physical activity in youth are characterized by brief periods of exercise of varying intensity interspersed with rest. To better understand systemic physiologic response mechanisms in children and adolescents, we examined five responses [heart rate (HR), respiratory rate (RR), oxygen uptake (V̇O_2_), carbon dioxide production (V̇CO_2_), and minute ventilation (V̇E), measured breath‐by‐breath] to multiple brief exercise bouts (MBEB). Two groups of healthy participants (early pubertal: 17 female, 20 male; late‐pubertal: 23 female, 21 male) performed five consecutive 2‐min bouts of constant work rate cycle‐ergometer exercise interspersed with 1‐min of rest during separate sessions of low‐ or high‐intensity (~40% or 80% peak work, respectively). For each 2‐min on‐transient and 1‐min off‐transient we calculated the average value of each cardiopulmonary exercise testing (CPET) variable (Y̅). There were significant MBEB changes in 67 of 80 on‐ and off‐transients. Y̅ increased bout‐to‐bout for all CPET variables, and the magnitude of increase was greater in the high‐intensity exercise. We measured the metabolic cost of MBEB, scaled to work performed, for the entire 15 min and found significantly higher V̇O_2_, V̇CO_2_, and V̇E costs in the early‐pubertal participants for both low‐ and high‐intensity MBEB. To reduce breath‐by‐breath variability in estimation of CPET variable kinetics, we time‐interpolated (second‐by‐second), superimposed, and averaged responses. Reasonable estimates of *τ* (<20% coefficient of variation) were found only for on‐transients of HR and V̇O_2_. There was a remarkable reduction in *τ*HR following the first exercise bout in all groups. Natural patterns of physical activity shape cardiorespiratory responses in healthy children and adolescents. Protocols that measure the effect of a previous bout on the kinetics of subsequent bouts may aid in the clinical utility of CPET.

## Graphical abstract

Natural patterns of physical activity shape cardiorespiratory responses in healthy children and adolescents. Protocols measure the effect of a previous bout on the kinetics of subsequent bouts may aid in the clinical utility of CPET.

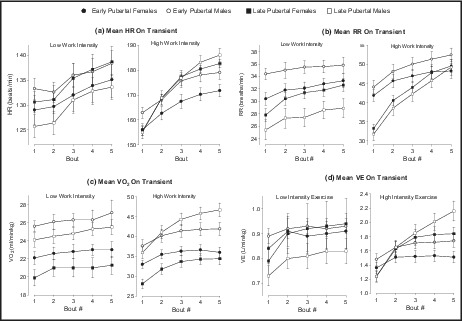

## 1 INTRODUCTION

The dynamic physiologic responses to patterns of physical activity when exercise starts and stops are increasingly seen as markers of cardiorespiratory and metabolic function in health and across a wide range of diseases (Buekers et al., 2020; Kiely et al., 2015; van de Vegte et al., 2019). The bulk of research on the kinetics of gas exchange flow [oxygen uptake (V̇O_2_), carbon dioxide production (V̇CO_2_), and minute ventilation (V̇E)] and frequency [heart rate (HR) and respiratory rate (RR)] variables associated with exercise has been done in adults. Although there is a growing body of novel insights in the pediatric population in whom, as in adults, disease tends to slow exercise responses, (Armstrong & Barker, 2009; Hebestreit et al., 2005; McNarry, 2019; Troutman et al., 1998) substantial gaps exist in our understanding of exercise responses in both healthy children and those with chronic disease. Most kinetic studies in children and adolescents utilize protocols adapted from adult studies, (Cooper et al., 1985; Lamarra et al., 1987) which do not take into account the way children and adolescents actually perform physical activity.

Natural patterns of physical activity (PA) in children are characterized by relatively short bursts (seconds to minutes) of exercise of various intensity interspersed with rest (Bailey et al., 1995; Gilliam et al., 1981). Multiple brief exercise bouts (MBEB), 2‐min bouts of constant work rate exercise interspersed by 1‐min of rest, was originally designed by our group (Radom‐Aizik et al., 2009) because many younger participants became bored with, fidgeted, varied pedaling rate on the cycle ergometer, and went on and off the mouthpiece during constant work rate exercise protocols that lasted more than a few minutes. MBEB was designed to test the dynamic effect of interval exercise intended to imitate more natural patterns of physical activity. There is evidence in adults that intermittent exercise is perceived as being less hard than the continuous exercise (Coquart et al., 2008). Because single bouts of exercise in children are brief, the overall response to a series of exercise bouts will be influenced by the inherent kinetic [on‐transient and off‐transient (recovery)] responses. This leads to the conclusion that the response of cardiopulmonary exercise testing (CPET) variables may differ over the course of an MBEB perturbation even when the work rate and duration of each individual exercise bout are constant and unchanged.

**FIGURE 1:**
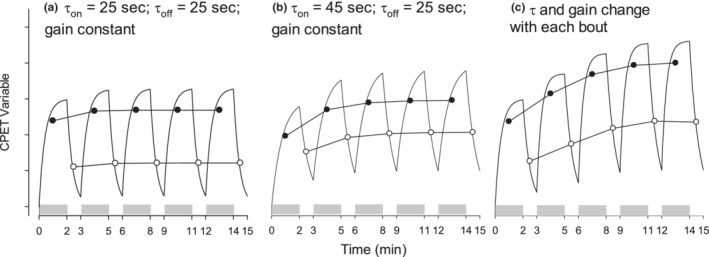
Theoretical effect of differences in time constant and gain on CPET variables in the first five bouts of MBEB (shaded areas). The modeled responses used the equations for the on‐ and off‐transients shown in the text. We calculated the effects of a few possible changes in *τ* and gain on the mean values (Y̅) of during on‐transients (closed circles) and off‐transients (open circles). Panel (a) shows the effect of a CPETvariable with *τ* = 25 sec and a constant (arbitrary) gain; panel (b), an increased *τ* and a constant gain; and panel (c), *τ* and gain increase with each bout. These theoretical data suggest that bout‐to‐bout changes in response characteristics might influence the mean values of CPET variables during bouts of exercise as occurs naturally in children and adolescents.

This concept is illustrated in Figure 1. The following simplified equations are often used to describe the on‐ and off‐transients of exercise responses to a constant‐work rate input.

(1) $$\begin{array}{c} \text{On‐transient}:Y_{t}=Y_{\mathrm{BL}}+A\left(1-e^{-\frac{t}{\tau }}\right), \end{array}$$

and

(2) $$\begin{array}{c} \text{Off‐transient}:Y_{t}=Y_{\mathrm{EX}}-A\left(1-e^{-\frac{t}{\tau }}\right), \end{array}$$

where $Y_{t}$ represents the CPET variable under analysis at any time (*t*); $Y_{\mathrm{BL}}$ is the baseline value of a given CPET signal; $Y_{\mathrm{EX}}$ is the value of the variable at the end of a given exercise bout; A is the amplitude or gain; and τ was the time constant of the exponential response of interest. The Figure illustrates the possible effects of different time constants and gains on a series of constant work rate exercise bouts. If, for example, an exercise bout during MBEB begins before substantial recovery from the previous bout has occurred, then the magnitude and pattern of the response values of the next bout will have been influenced by the previous one. The figure highlights the many ways in which the physiological responses to a series of exercise bouts can change from bout‐to‐bout.

Maturational status and sex play roles in the kinetics of CPET variables. There is evidence, for example, that healthy, early‐pubertal children have substantially faster HR, V̇E, and V̇CO_2_ exercise responses than healthy late‐pubertal or adult individuals (Baraldi et al., 1991; Cooper, Kaplan, Baumgarten, et al., 1987). V̇O_2_ kinetics appear to be less dependent on maturational status, but children have higher oxygen uptake per work performed (gain) than do late‐pubertal or adult individuals (Armon, Cooper, Flores, et al., 1991; Zanconato et al., 1991a). Work intensity also influences on‐transient and recovery dynamics (Lucrezia et al., 2018). Consequently, as the work intensity of an individual exercise bout increases, the gain and time constants change, and first‐order linear system equations may not be sufficiently comprehensive to describe on‐ and off‐transient dynamics in MBEB. Consistent with this, seminal work by Barker et al. (2010, 2014) demonstrated that for high‐intensity exercise in children, 6‐minute priming exercise led to faster V̇O_2_ kinetics in subsequent bouts of high‐intensity exercise.

The goal of this study was to characterize and quantify the gas exchange and frequency responses using an exercise protocol designed to mimic real‐life PA in a cohort of healthy early‐ and late‐pubertal boys and girls.

Focusing on two CPET physiological frequency variables, HR and RR, and three CPET flow variables, V̇O_2_, V̇CO_2_, and V̇E, we tested the following hypotheses in healthy children:

1. In MBEB protocol, CPET variable responses to exercise change from bout to bout.
2. MBEB CPET variables will be influenced by maturational status, sex, and the relative magnitude of the exercise input (the relative exercise intensity).

Participants were categorized into the following eight subgroups for the purpose of stratified analyses: early‐pubertal females at (1) low‐work intensity MBEB (40% of peak work) and (2) high‐work intensity (80% of peak work); early‐pubertal males at (3) low‐work intensity and (4) high‐work intensity; late‐pubertal females at (5) low‐work intensity and (6) high‐work intensity; and late‐pubertal males at (7) low‐work intensity and (8) high‐work intensity.

## 2 METHODS

**FIGURE 2:**
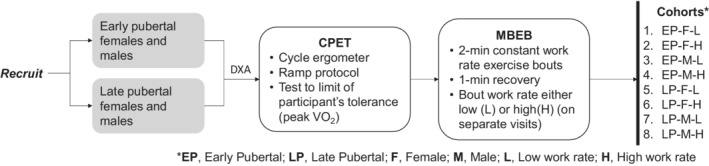
Overview of the study.

A schematic overview of the study is shown in Figure 2. The protocol was approved by the UC Irvine Institutional Review Board. Informed consent was obtained from legally authorized guardians and, where appropriate, assent from the participants themselves. Inclusion criteria included healthy 7–18 y.o. participants without any known respiratory, cardiac, or metabolic disease and not taking any chronic prescribed medication. Body mass index (BMI) of each participant was less than the 95th percentile (BMI Calculator Child and Teen, n.d.). Each volunteer visited the laboratory on three occasions. During the first visit, informed consent was obtained, and demographic and anthropometric data were recorded, Tanner stage (by questionnaire; height, skin changes, pubic hair and, for boys: facial hair and voice changes and for girls: breast development and menses) was assessed, (Petersen et al., 1988) and dual X‐ray absorptiometry (DXA) was performed. A maximal progressive exercise protocol on a cycle ergometer (CE) was also done on the first visit. At least 48 h later, each participant completed two separate visits in random order: low (40% of peak work rate) and high (80% of peak work rate) MBEB.

### 2.1 Analytic approach

**FIGURE 3:**
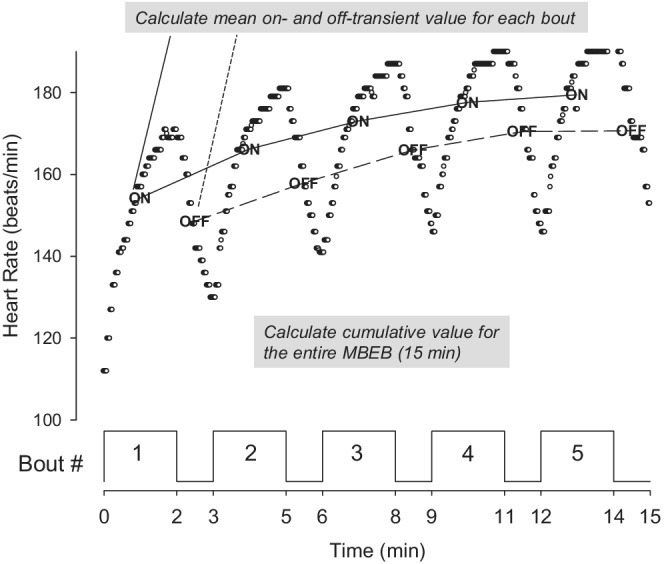
Model‐agnostic CPET analysis.

Breath‐to‐breath variability poses a challenge to the simplification and summary of CPET analyses, noted, for example, in the many diverse approaches to the determination of V̇O_2_ max or peak (Neder et al., 2021). An additional obstacle to analysis is that breath‐to‐breath variability is greater in children compared with adults (Potter et al., 1999). As we have done in earlier studies (Armon, Cooper, & Zanconato, 1991; Zanconato et al., 1991a), we mitigated breath‐by‐breath variability in several ways using model‐agnostic and model‐dependent approaches to data analysis. Model agnostic approaches impose no assumptions on the data in contrast to model‐dependent data analyses. For example, attempting to calculate the time constant from a dataset assumes an exponential model, whereas calculating an average value of V̇O_2_ or V̇CO_2_ over the course on an exercise bout does not. To assess whether CPET variables changed from bout to bout, we calculated the average value of each CPET variable for each bout in each participant (Equation 3, Figure 3).

(3) $$\bar{Y}=ƩY_{t}÷t_{\text{bout}},$$

where Y̅ is the average value for Y (HR, RR, V̇O_2_, V̇CO_2_, or V̇E) and t_bout_ was 120 s for the on‐transients and 60 s for the off‐transients.

We also calculated the cumulative responses of the CPET variables over the entire 15 min of the MBEB (Equation 4).

(4) $$\begin{array}{c} \text{Cumulative}\;\left(t_{0}\;\text{to}\;t_{15\;\min}\right)\;\text{response}\;\left(t_{0}\;\text{to}\;t_{15\;\min}\right)\\ =ƩY/Ʃ\;\text{work rate}, \end{array}$$

where Y represented the CPET flow variables, V̇O_2_, V̇CO_2_, or V̇E.

**FIGURE 4:**
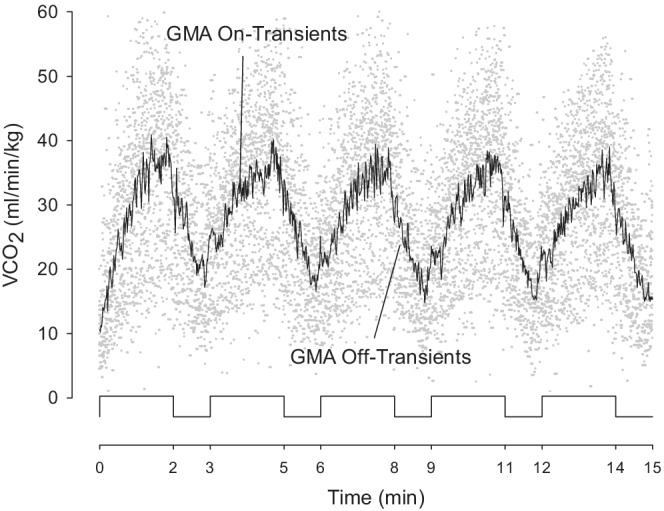
Time‐interpolated group mean average to determine *τ* and amplitude.

Exercise response kinetics involves estimating the time constant (*τ*) and gain of various forms of exponential equations, and is accomplished by iterative curve fitting. Attempts to determine time constants by curve fitting from individual bouts in adults and even more so in children typically result in nonconvergent or unacceptably large estimate errors. In a seminal study in adults (Lamarra et al., 1987), recognized the need to minimize breath‐by‐breath variability in order to obtain reasonably accurate estimates of *τ*. Since individual breaths vary in duration, Lamarra introduced the approach of time‐interpolation of breath‐by‐breath data so that the results from individual participants could be superimposed and averaged second‐by‐second. Lamarra used up to eight repeated constant work rate exercise tests per subject, each lasting 5 min, to produce reasonably accurate estimates of the on‐ and off‐transient time constants. These were obtained in highly cooperative young adults, willing to repeat constant work rate exercise tests on numerous occasions. Lamarra et al. noted that the estimate of *τ* and its confidence interval depends in a complex fashion on the noise‐to‐signal ratio (as noted, known to be greater in children compared with adults Potter et al., 1999), and the amount of data available for fitting. As demonstrated in Figure 4, we applied the approach of time‐interpolation and averaged the second‐by‐second responses of each participant in the eight experimental groups (group mean average, GMA).

We estimated *τ* of each of the five MBEB bouts from the GMA data of HR, RR, V̇O_2_, V̇CO_2_, and V̇E using Equations 1 and 2 shown above. To determine best fit, a nonlinear algorithm of least squares was used. It adopts the minimization of the sum of squared errors as a criterion for convergence and calculates the *τ* estimate ± SEM.

### 2.2 Participants (Table 1)

**TABLE 1:** Anthropometric and peak VO_2_ in study participants

Cohort	N	Age (year)	Weight (kg)	Height (cm)	BMI (%tile)	LBM (kg)	Fat (%)	PeakV̇O_2_ (ml/min/kg)
EPF	17	9.0 ± 1.2	29.7 ± 7.7	131.9 ± 9.1	49.8 ± 28.1	19.0 ± 3.7	32.8 ± 5.5	44.9 ± 7.1
EPM	20	10.7 ± 1.8	36.7 ± 11.8	143.6 ± 12.2	45.1 ± 29.9	24.9 ± 6.4	28.5 ± 5.9	52.5 ± 7.0
LPF	23	15.3 ± 1.6	55.1 ± 8.4	161.2 ± 6.0	57.0 ± 23.6	35.7 ± 4.5	32.3 ± 5.0	41.7 ± 7.0
LPM	21	17.0 ± 1.4	62.6 ± 10.3	171.7 ± 7.3	45.2 ± 25.0	46.4 ± 7.6	23.2 ± 6.7	52.7 ± 11.4

Abbreviations: BMI, Body mass index; EPF, early‐pubertal females; EPM, early‐pubertal males; LBM, lean body mass; LPF, late‐pubertal females; LPM, late‐pubertal males; V̇O_2_ – oxygen uptake.

Eighty‐one participants were recruited. As shown in Table 1, the participants were equally distributed. The volunteers reflected the racial and ethnic composition of our region [Caucasian 75 (93%), Hispanic/Latino 4 (5%), and African American 2 (2%)]. All participants were screened and determined to be healthy based on interviews to identify any congenital or chronic diseases and conditions that would impair physiological responses to exercise. Extremely physically active participants (e.g., those considered to be elite athletes involved in routine intensive exercise training) were also excluded. Each participant performed MBEB at low and high constant work rates on separate occasions.

Standard, calibrated scales and stadiometers were used to determine total body mass and height. Body composition, including lean body mass (LBM), was determined by DXA using a Hologic QDR 4500 densitometer (Hologic Inc.). Participants were scanned in light clothing while lying supine. On the day of each test, the DXA instrument was calibrated using the procedures provided by the manufacturer, and DXA scans were performed and analyzed using pediatric software. As noted, a commonly used self‐assessment questionnaire for population studies was used to assess pubertal status quantified as early pubertal (Tanner 1–2) and late pubertal (Tanner 4–5).

### 2.3 Exercise testing

The study consisted of three separate exercise testing sessions completed over a course of no more than 12 weeks. The first session consisted of a standardized, ramp‐type progressive exercise test in which the participant pedaled on a cycle ergometer until they reached the limit of tolerance (Cooper et al., 1984). Gas exchange was measured breath‐by‐breath using the SensorMedics metabolic system (Vmax Encore 229). Participants were vigorously encouraged during the high‐intensity phases of the exercise protocol. Peak V̇O_2_ was determined when RER ≥1.0 and was calculated as the highest 20‐s rolling average in the last minute of exercise (Rowland et al., 2008). The results of the ramp CPET were then used to set the individualized baseline work rate for the subsequent MBEB session scheduled for separate days. MBEB consisted of up to 10 2‐min bouts of constant work rate exercise on a CE with a 1‐min rest period after each bout. Study visits were scheduled between the morning and early afternoon and participants were asked to abstain from heavy exercise beginning on the day prior to the MBEB test.

The work rate MBEB was calculated for each participant as low‐intensity work rate, 40% of peak work rate (below the lactate/anaerobic threshold [LAT]), and high‐intensity work rate, 80% of peak work rate. The MBEB protocols were performed on different days and in random order (sessions 2 and 3). No warm‐up exercise was performed. After each 2‐min bout, the participants were instructed to affirm their willingness to continue on with the next bout. For both the low‐ and high‐intensity exercise sessions, we asked each participant to try to complete 10 bouts of exercise in the MBEB. However, we found that during the high‐intensity MBEB, 50% of the participants were unable to complete 10 bouts, while all participants successfully completed five bouts. Thus, we analyzed the data for the first five bouts of the MBEB for both low‐ and high‐intensity work rates.

### 2.4 Scaling and normalization

As previously published, (Cooper et al., 2014) CPET gas exchange variables are highly body mass dependent since V̇O_2_, V̇CO_2_, and V̇E are ultimately driven by energetic demand in the muscle tissue. In contrast, frequency variables (HR and RR) are less dependent on body mass. The role of developmental and allometric factors remains the topic of lively and constructive debate, (Armstrong & Welsman, 2020; Cooper & Berman, 1994) but no widely accepted standardization yet exists for comparing CPET in children, adolescents, and adults in whom body mass and developmental stage vary widely. In comparing body weight to the DXA‐derived lean body mass estimate, we found a correlation (*r*) of 0.97, providing strong evidence to use body weight as a surrogate for muscle mass in scaling size‐dependent CPET variables (Cooper et al., 2014). For the cumulative analyses of CPET variables over the 15‐min MBEB, we scaled V̇O_2_, V̇CO_2_, and V̇E to the work performed (watts), an approach that relates the physiologic response to a thermodynamically incontrovertible unit of energy expenditure.

### 2.5 Statistical analysis

For each CPET variable, we examined the changes in on‐transient and off‐transient response from Bout 1 to Bout 5 in each group. Stratified linear mixed‐effects regression models were developed to evaluate these bout‐to‐bout trends, accounting for repeated measures while allowing for between‐subject variation through random slopes and intercepts. Post hoc polynomial contrasts yielded by these models, allowed us to evaluate the presence of a linear trend across the bout means (measured by the linear contrast t‐ratio) for each subgroup. A linear contrast t‐ratio >0 indicates a positive linear relationship between the response variable and the bout sequence number; likewise, *t*‐ratio < 0 indicates an inverse relationship. An absolute value of *t*‐ratio > 1.96 indicates statistical significance.

We used a different approach for the cumulative CPET variables, those obtained over the 15‐min MBEB data. In this analysis, generalized linear regression models were performed to test for sex and maturational main effects as well as their interaction within each intensity level.

For the estimates of *τ* from the GMA data, we did not apply formal tests of statistical significance, but rather present the key data as the estimate of *τ* from the curve fitting and its SEM. Within each sex‐puberty‐intensity subgroup, estimated paired comparison effects sizes were calculated comparing Bout 1 to Bout 5 as well as Bout 1 to the average of Bouts 1–5. An estimated intercorrelation of 0.6 was used for these calculations.

## 3 RESULTS

The MBEB protocol revealed significant bout‐to‐bout changes in 67 of 80 on‐ and off‐transients.

### 3.1 Bout‐to‐Bout mean values

**FIGURE 5:**
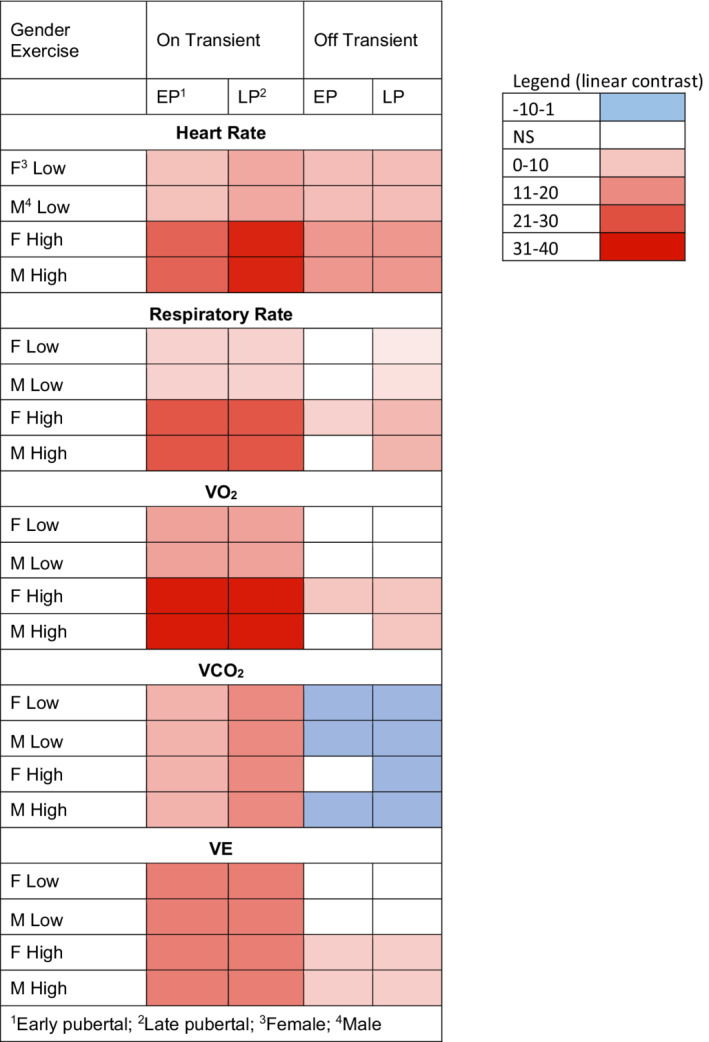
Heat map of linear contrast *t*‐ratio statistic of the selected CPET variables in the on‐ and off‐transients of the eight groups. The linear contrast values indicate the relative statistical strength of the trend. A higher value (dark red) indicates a strong positive linear trend from Bout 1 to Bout 5, while values <0 indicate a negative trend (blue).

**FIGURE 6:**
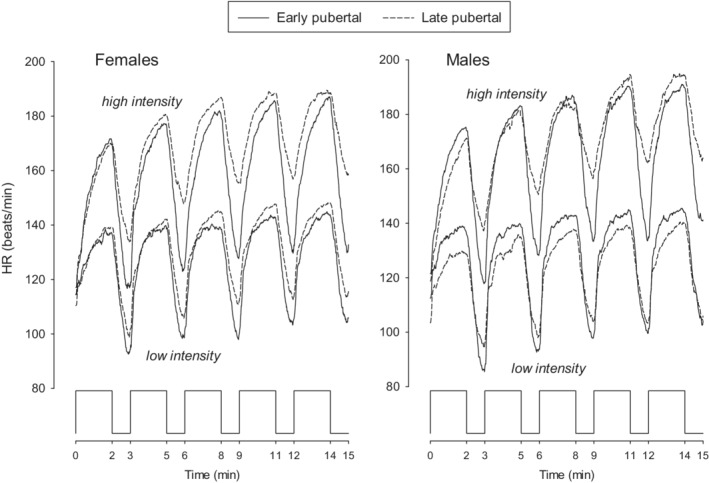
GMA bout‐to‐bout HR responses to the MBEB protocol in all eight study groups.

**FIGURE 7:**
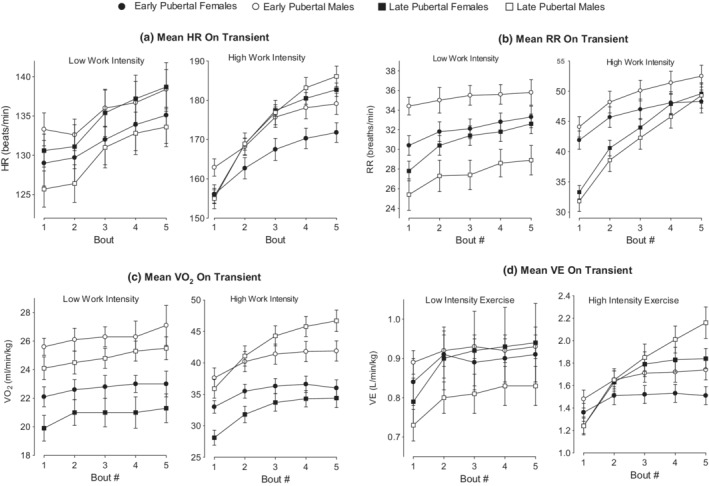
Summary of mean bout‐to‐bout HR, RR, V̇O_2_, and V̇E during on‐transients; each of these variables increased from bout to bout.

**FIGURE 8:**
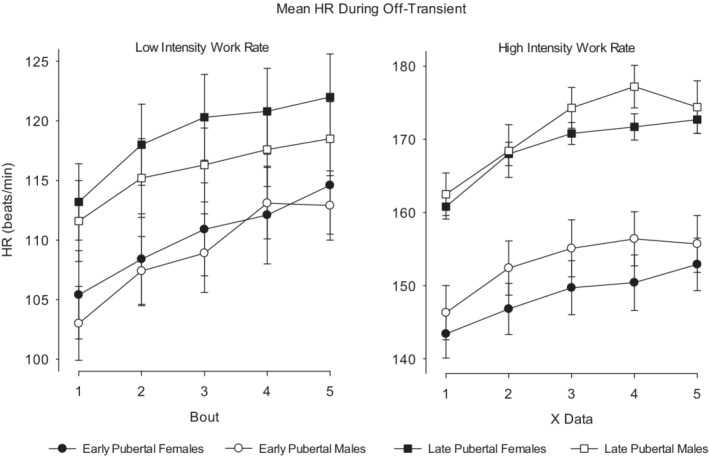
Off‐transient mean HR values. The consistently higher off‐transient HR values correspond to the GMA data shown in Figure 6. In the early‐pubertal participants, HR returned to baseline much more quickly than in the late‐pubertal participants.

Bout‐to‐bout Y̅ increased in MBEB for all on‐transient CPET variables (HR, RR, V̇O_2_, V̇CO_2_, V̇E) in both females and males, at both low‐ and high‐work rates, and in both pre‐ and late‐pubertal participants (Figures 5, 6, 7, 8). The bout‐to‐bout increases were significantly greater during the on‐transient, high‐intensity MBEB for HR, RR, and V̇O_2_ (Figure 5) than in the low‐intensity MBEB. For example, in low‐intensity exercise, Y̅_V̇O2_ from Bouts 1 to 5 increased significantly in early‐pubertal males from 25.6 ± 0.6 to 27.1 ± 1.4 ml/min/kg and in late‐pubertal males from 24.1 ± 0.8 to 25.5 ± 0.8 ml/min/kg. In high‐intensity MBEB, the increase was from 37.6 ± 1.6 to 41.9 ± 1.6 ml/min/kg in the early‐pubertal males, while in the late‐pubertal males, the increase was from 35.9 ± 1.5 to 46.7 ± 1.7 ml/min/kg. Off‐transient mean bout‐to‐bout values were less consistently affected, but the off‐transient HR increased in all groups.

### 3.2 Cumulative CPET MBEB results

**FIGURE 9:**
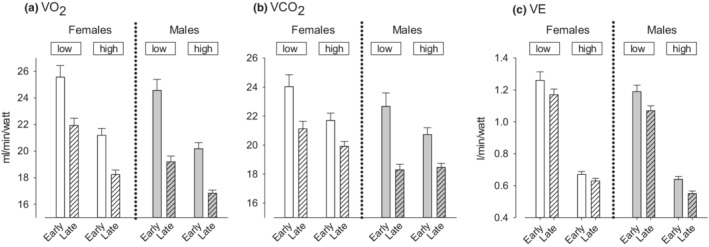
Mean MBEB gas exchange variables scaled to work rate calculated over the 15‐min protocol for V̇O_2_ (a), V̇CO_2_ (b), and V̇E (c). When analyzing low‐intensity MBEB as a group in males and females, early‐pubertal participants had significantly higher values than late‐pubertal participants for V̇O_2_ (*p* < 0.0001), V̇CO_2_ (*p* < 0.0001), and V̇E (*p* = 0.01). For high‐intensity MBEB, early‐pubertal participants had significantly higher values than late‐pubertal participants for V̇O_2_ (*p* < 0.0001), V̇CO_2_ (*p* < 0.0001), and V̇E (*p* = 0.0002). When analyzed as a group, females during low‐intensity MBEB had higher values than males for V̇O_2_ (*p* < 0.0081), V̇CO_2_ (*p* < 0.0037), and V̇E (*p* = 0.0044); females during high‐intensity MBEB had higher values than males for V̇O_2_ (*p* < 0.0032), V̇CO_2_ (*p* < 0.0041), and V̇E (*p* = 0.0036). We found no significant sex × pubertal stage interaction.

1. V̇O_2_ cumulative values. We found significantly greater costs of MBEB normalized to the work performed over the 15‐min period in early‐pubertal compared with late‐pubertal participants and for low‐ compared with high‐work intensity MBEB (Figure 9).
2. V̇CO_2_ cumulative values. We found significantly greater values in early‐pubertal compared with late‐pubertal participants. Low‐ compared with high‐work intensity cumulative V̇CO_2_ was greater in low‐intensity work with the exception of the late‐pubertal males.
3. V̇E cumulative values. We found significantly greater values in early‐pubertal compared with late‐pubertal participants. Unlike V̇O_2_ and V̇CO_2_, no consistent effect of work intensity was observed.
4. The cumulative values of V̇O_2_, V̇CO_2_, and V̇E were significantly higher in females compared with males.

### 3.3 Time constants

**FIGURE 10:**
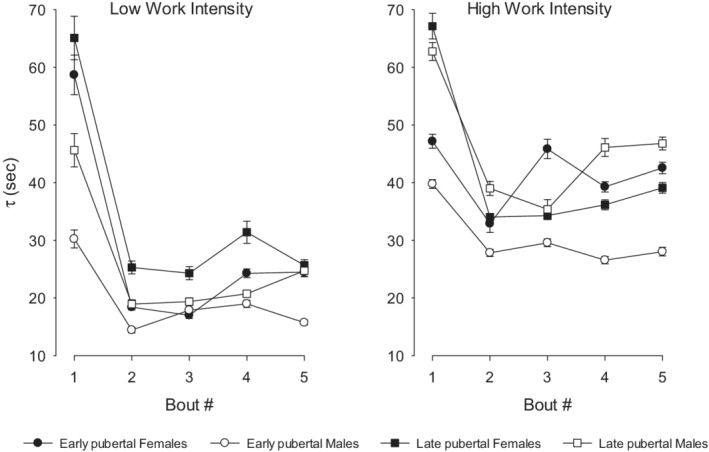
On‐transient *τ* HR, there was a substantial decrease in *τ* HR for all comparison groups following the first bout.

Based on previous estimates of time constants, we excluded estimates that were likely to be artifacts (i.e., physiologically unreasonable, namely, ≥100 s and coefficient of variation >20%). Our calculation of *τ* from GMA revealed that only the on‐transients of HR and V̇O_2_ met these criteria. As shown in Figure 10, we found a remarkable reduction in *τ*HR following Bout 1. No distinct patterns in *τ*HR were observed for Bouts 2–5 in any of the groups. We did not observe consistent group or bout‐to‐bout changes for *τ*V̇O_2_. For *τ*HR, higher‐work intensities were associated with longer time constants in all groups. No consistent patterns were observed for *τ*V̇O_2_.

## 4 DISCUSSION

These data demonstrate the substantial and dynamic effect of brief, repeated, constant‐work rate bouts of physical activity on essential physiological responses to exercise in children and adolescents. The results suggest that laboratory protocols designed to mimic real‐life physical activity can yield novel insights into maturational and work intensity mediators of CPET variables. In a series of short bouts of exercise interspersed with brief rest, individual bouts can influence the gas exchange and frequency of the subsequent on‐ and off‐transients of the subsequent bouts. The data indicate that brief exercise leads to dynamic physiologic adjustments that can alter the metabolic cost, as well as the frequency and gain of subsequent bouts.

There has been much investigation demonstrating the effect of relatively brief bouts of exercise (warm‐up exercises) on subsequent physiological exercise responses (e.g., the review by McGowan et al., 2015). The bulk of these studies were done predominantly in adults with very few reports in children and have been aimed at identifying strategies, often quite complex, to improving performance in athletic events and competitions and preventing injuries (Thorborg et al., 2017). The ultimate effect, either improved (Silva et al., 2018) or impaired (Christensen & Bangsbo, 2015) subsequent performance, appears to depend on the type, duration, and intensity of the proposed warm‐up, as well as the particular athletic competition or event for which the warm‐up was implemented. Given these varied effects of warm‐ups, current CPET protocols, even those with a specified pre‐test warm‐up, typically involve continuous increases in work intensity, and, consequently, may not reveal dynamic responses reflecting adjustments to the work perturbation that can influence subsequent exercise.

Our data suggest that relatively brief constant work rate exercise bouts are sufficiently substantial to increase mean values of HR, RR, V̇O_2_, V̇CO_2_, and V̇E in the subsequent MBEB bouts. Even in the brief off‐transients, bout‐to‐bout increase in HR was observed as well. In general, the magnitude of the bout‐to‐bout effects was larger for high‐intensity exercise, consistent with studies of high‐intensity exercise in both children and adults, (Haouzi et al., 1993; Lambrick et al., 2013a; Lucrezia et al., 2018) revealing a slow component upward rise in V̇O_2_ even when the work rate is constant. In the high‐intensity work rate MBEB, the magnitude of increase in the Bouts 1–5 mean V̇O_2_ was much greater in the late‐pubertal participants than the increase observed in the early‐pubertal participants (Figures 6 and 7). The whole‐organism, systemic data obtained during CPET cannot be used to precisely determine which tissue‐level interactions in any single bout of the MBEB protocol were most responsible for the effects on subsequent bouts. Each of the CPET variables that we measured is mediated by a complex control system including autonomic nervous activation, circulating mediators such as pH and catecholamines, and possibly cognitive factors. Nonetheless, given the critical and rapidly responsive systemic effects of tissue oxygen delivery, we speculate that V̇O_2_ and its kinetics are likely the main controlling signal for the integrated systemic response and initiate the changes in HR and the remaining gas exchange variables.

The physiological mechanisms responsible for the slow component upward rise in V̇O_2_ referred to above have, in recent years, undergone renewed scrutiny. Muscle critical power (CP), determined in part by oxidative phosphorylation activity, is related to V̇O_2_ kinetics (Korzeniewski & Rossiter, 2021). The CP concept describes the work rate (power) above which the duration of sustained exercise rapidly diminishes (Poole et al., 2016). The high‐intensity MBEB used in this study, based on data obtained from the ramp protocols, likely placed each participant above the CP range of exercise. Korzeniewski and Zoladz (2015) suggested both the progressive inhibition of anaerobic glycolysis by accumulating protons and gradual increase in ATP usage during heavy exercise contributed to the observed upward slope of V̇O_2_ during high‐intensity MBEB. Interestingly, (Davies et al., 2017) have also suggested that intermittent bouts of exercise compared to constant work rate improves aerobic capacity through greater usage of stored O_2_ in intermittent exercise. High‐intensity intermittent exercise has been shown to improve training in adults and more recently in children (Lambrick et al., 2013b; McNarry et al., 2015). The bout‐to‐bout increases in V̇O_2_ during high‐intensity exercise are consistent with current understanding of the slow component, and our results may ultimately prove useful in designing effective intermittent exercise testing and training regimens for children and adolescents in both health and disease.

We also found small increases in mean bout V̇O_2_ and the other CPET variables during low‐intensity exercise. In our study, the work rates for these MBEB CPETs were likely below the CP and not likely to have led to the compensatory mechanisms that drive an increased V̇O_2_ in the higher‐intensity MBEB. The increases in bout average values may have resulted from the mechanism outlined in Figure 1, namely, that the 2‐min exercise and 1‐min recovery were not of sufficient duration for the physiological systems to reach a steady state when gas exchange is measured at a distance from the exercising muscle (the primary tissue responsible for O_2_ uptake and CO_2_ production). Consequently, the transit times from muscle to breath, which are influenced by a variety of factors, such as circulatory response and systemic tissue and blood storage of O_2_ and CO_2_, indicate that breath‐by‐breath measurements in subsequent MBEB bouts include gas exchange remnants from the previous exercise. While we recognize that first‐order exponential equations are likely oversimplifications, they do demonstrate that both the response time and the amplitude (gain) ultimately contribute to the average value in any given MBEB bout. As noted in Figure 1, the changes from one bout to another can result from changes in the subsequent time constants and gain or, alternatively, because of complex interactions of ongoing transitory signals from the previous bout.

While the pattern of bout‐to‐bout change was similar for the remaining frequency and flow CPET variables, there were differences that revealed the effect of maturational state. For example, mean bout RR was higher in bouts 1–4 in the early‐pubertal participants during high‐intensity MBEB, but the increase in the late‐pubertal participants was so rapid that by Bout 5, RR in all cohorts was virtually indistinguishable (Figure 7). We also observed a substantial maturational effect in HR, the other frequency CPET variable. As shown in Figures 6 and 8, HR recovery in the early‐pubertal participants was much more complete than in the late‐pubertal groups. RR is regulated by V̇CO_2_, and the pCO_2_, the CO_2_ “set point,” and anatomic factors such as the relationship between deadspace and lung volume (Armon et al., 1990; Cooper, Kaplan, Baumgarten, Weiler‐Ravell, et al., 1987; Pearsall & Feldman, 2014)—all mechanisms that change during growth. RR is determined by both autonomic and cognitive control mechanisms, while HR is predominantly autonomic, but can clearly be influenced by environmental factors like emotional stress. HR recovery appears to be predominantly controlled by autonomic factors whatever the initial perturbation may have been, (Pierpont et al., 2013) and, not surprisingly, is more consistent than RR in response to exercise (Barrera‐Ramirez et al., 2013). Very little research has been done to gauge the effect of maturational status on HR kinetics in response to exercise, but earlier work does suggest more rapid response times in younger children, particularly following heavier exercise (Baraldi et al., 1991; Buchheit et al., 2010; Ratel & Blazevich, 2017; Zanconato et al., 1991a). Whether the more rapid initial RR response to exercise resulted from an accentuated effect of environmental factors, perhaps the excitement of starting the exercise test, has yet to be determined.

The cumulative data also shed light on maturational differences in the response to the MBEB protocol (Figure 9). The V̇O_2_ costs were higher for early‐pubertal participants and were higher in the low‐ compared with the high‐work intensity. Earlier research has demonstrated a great O_2_ dependence for work, thought to be due to less utilization of anaerobic metabolic pathways in the muscle in early‐pubertal children compared with adults, a result consistent with generally lower levels of circulating lactate concentrations during exercise in children (Chen et al., 2018; Zanconato et al., 1991b). A greater dependence on aerobic metabolism would also explain the larger V̇CO_2_ and V̇E responses that we observed. Increased oxidative phosphorylation in the younger participants will lead to increases in both O_2_ consumption and CO_2_ production, and the latter will drive V̇E. The generally higher values for the gas exchange variables in males compared with females are most likely due to the larger muscle mass as well as larger V̇O_2_ per body weight observed in the males.

We did observe larger V̇O_2_, V̇CO_2_, and V̇E costs of MBEB in females compared to males (n.b., there was no sex × pubertal stage interaction). In the case of V̇O_2_, it has been challenging to directly compare oxidative capacity in response to exercise between women and men due to factors such as differences in body composition, (Diaz‐Canestro et al., 2021) blood volume, (Diaz‐Canestro et al., 2022) distribution of muscle fiber types, (Fournier et al., 2022) and gauging overall levels of conditioning. Men may demonstrate greater V̇O_2_ responsiveness to exercise training than do women, (Diaz‐Canestro & Montero, 2019; Montero et al., 2018) suggest that even though muscle mass is smaller in women, mitochondrial volume is greater, perhaps explaining our finding of great V̇O_2_ costs when normalized to the work performed. Recently, (Wilkinson & Shirwa, 2021) suggested the intriguing hypothesis that at work rates above critical power, muscles in females exhibited greater uncoupling of oxidative phosphorylation than men, which might also explain great V̇O_2_ per work rate than in males. In the pediatric literature, (Armstrong et al., 2015) reviewed a large series of studies and concluded that relatively larger peak V̇O_2_ (scaled allometrically to body mass) became evident only in males during young adulthood. Finally, sexual dimorphism in respiratory control in adults is a known phenomenon, but the impact of sex on respiratory control during exercise in children and adolescents is not well understood. In a recent study, higher ratio of V̇E to V̇CO_2_ during exercise was observed in females (a group of children and young adults) than in males (Bar‐Yoseph et al., 2019). Clearly, more studies are needed to better understand the sexual dimorphism we observed in MBEB in children and young adults.

The challenges associated with accurate determination of model‐fitting determination in the highly variable data produced in breath‐by‐breath measurements of gas exchange during MBEB prompted us to use noise‐reducing analytic techniques. We chose to look at the overall (averaged or cumulative) costs of frequency and gas exchange variables over the 15‐min of MBEB. Despite these approaches, only the on‐transients of HR and V̇O_2_ met our acceptability criteria (*τ* estimates <100 s and coefficient of variation ≤20%). The HR data showed a remarkable reduction in *τ* following the initial bout in all groups, in both low‐ and high‐intensity exercise. All participants were at rest for at least 40 min prior to the onset of the MBEB. We speculate that the single exercise bout, whether low‐ or high‐intensity, was sufficient to alter the sympathetic and parasympathetic balance for the remaining exercise bouts.

### 4.1 Study challenges

This project was designed as a feasibility study. Although the initial data analysis encourages further exploration of novel approaches to exercise testing in children, challenges to greater generalization and additional use need to be overcome. In addition, equations # 1 and 2 are simplified representations of the kinetics, and future studies may demonstrate more accurate models. The construction of our MBEB protocol, in particular with only 1‐min allotted to recovery following each exercise bout, was likely to lead to incomplete recovery given current knowledge of gas exchange and HR kinetics in children and adolescents. Additional studies are needed to determine the bout‐to‐bout effects with different intervals of work rate on‐ and off‐transients. Nonetheless, natural patterns of physical activity and rest in‐between are brief, suggesting that bout‐to‐bout changes whether they result simply from incomplete recovery or from more substantial changes in the cellular and systemic responses to the exercise input, may play a meaningful role in exercise behavior. Our participant population, while homogenous and reflective of the local community at our site, was not representative of the population as a whole and further studies will be necessary to gauge the effect of racial, ethnic, and other social determinants on exercise responses as children grow and develop. We did not include obese children or adolescents (BMI%ile > 95%), and given the clear impact of childhood obesity on exercise responses (Cooper et al., 2016) as well as the increased lifelong increase in cardiovascular disease risk, (McPhee et al., 2020) future studies should focus on this population. Successful recruiting of relatively large numbers of children and adolescents must account for busy school schedules, transportation issues, and availability of parents and authorized caregivers. We scheduled participants at their convenience and did not attempt in the present study to account for possible effects of circadian rhythm (Hill, 2014). We did request that participants refrain from exercise for the day prior to MBEB testing, which was verified by interview but not by remote activity monitoring.

## 5 CONCLUSIONS

These data add to an emerging understanding of exercise in which physiological responses adjust not only to affect sufficient gas exchange to sustain the immediate work rate challenge, but possibly to prepare the organism for subsequent exercise bouts. Bout‐to‐bout changes in frequency and gas exchange variables were found in both low‐ and high‐intensity MBEB. Not surprisingly, the magnitude of these changes was greater in the high‐intensity MBEB, where increases in lactate, catecholamines, and other inflammatory mediators are known to rise in the circulation. Both the bout‐to‐bout and overall response to MBEB differed between early‐ and late‐pubertal participants, highlighting the role of normal growth and development of the cardiorespiratory, neuroendocrine, and vascular systems in modulating the exercise response.

There is robust and emerging interest in exercise training paradigms for children and adolescents that more closely mimic more natural physical activity, (e.g., intermittent high intensity) highlighting the value of more thorough understanding of exercise kinetic responses during growth and development. In addition, kinetic responses to exercise are increasingly seen as sensitive indicators of fitness in health and disease in both children and adults. Consequently, approaches like the MBEB may prove useful in developing innovative solutions to physical fitness assessment across the lifespan.

## AUTHORS' CONTRIBUTIONS

Ronen Bar‐Yoseph MD. Directed and managed all aspects of the project including hypothesis generation, study design, data acquisition, data analysis, and interpretation. Shlomit Radom‐Aizik PhD. Participated and collaborated with all aspects of the project including hypothesis generation, study design, data acquisition, data analysis, interpretation, discussion, and manuscript preparation and writing. Nicholas Coronato. Participated in hypothesis generation, data curation and analysis, and manuscript preparation and writing. Nazanin Moradinasab. Participated in hypothesis generation, data curation and analysis, and manuscript preparation and writing. Thomas J. Barstow PhD. Participated in hypothesis generation, critical concept review, and manuscript preparation and writing. Annamarie Stehli. Participated and collaborated with all aspects of the project including hypothesis generation, study design, data acquisition, data analysis, interpretation, discussion, and manuscript preparation and writing. Don Brown PhD. Participated and collaborated with all aspects of the project including hypothesis generation, study design, data acquisition, data analysis, interpretation, discussion, and manuscript preparation and writing. Dan M. Cooper MD. Participated and collaborated with all aspects of the project including hypothesis generation, study design, data acquisition, data analysis, interpretation, discussion, and manuscript preparation and writing.

## CONFLICT OF INTEREST

None of the authors have conflicts of interest.
